# Significance of Visual Evoked Potentials in the Assessment of Visual Field Defects in Primary Open-Angle Glaucoma: A Review

**DOI:** 10.1155/2013/418320

**Published:** 2012-12-06

**Authors:** Ruchi Kothari, Pradeep Bokariya, Smita Singh, Ramji Singh

**Affiliations:** ^1^Department of Physiology, Mahatma Gandhi Institute of Medical Sciences, Sevagram, Wardha 442102, India; ^2^Department of Anatomy, Mahatma Gandhi Institute of Medical Sciences, Sevagram, Wardha 442102, India; ^3^Department of Ophthalmology, Mahatma Gandhi Institute of Medical Sciences, Sevagram, Wardha 442102, India

## Abstract

Visual evoked potentials is an important visual electrophysiological tool which has been used for the evaluation of visual field defects in primary open-angle glaucoma and is an appropriate objective measure of optic nerve function. Significant correlations between the magnitude of the VEP parameters and MD of Humphrey static perimetry suggest that the impaired visual cortical responses observed in glaucoma patients can be revealed by both electrophysiological and psychophysical methods. In addition, the severity of global glaucomatous damage evidenced by reduction in MD could depend on the delay in neural conduction from retina to the visual cortex as revealed by the significant correlation between VEP latencies and MD which also supports the validity of the VEP testing in progression of glaucoma.

## 1. Introduction

Primary open-angle glaucoma is described distinctly as a multifactorial optic neuropathy that is chronic and progressive with a characteristic acquired loss of optic nerve fibers. Such loss develops in the presence of open anterior chamber angles, characteristic visual field abnormalities, and intraocular tension that is too high for the continued health of the eye. It manifests by cupping and atrophy of the optic disc, in the absence of other known causes of glaucomatous disease. 

Thus the clinical diagnosis of POAG is commonly based on increase in intraocular pressure, characteristic optic nerve head cupping, and typical visual field defects which are assessed by standard static threshold perimetry, using an automated system such as Humphrey field analyzer (HFA). HFA however does not selectively reveal which structures contribute to the impairment of the visual system observed in glaucoma. It has been suggested that damage to the ganglion cells and/or their axons produce glaucomatous visual field defects. In this context, electrophysiological testing provides specific and unique information. Electrophysiological tests like visual evoked potentials can contribute to detection of glaucomatous optic neuropathy since they are compatible with the functions of retinal ganglion cells, and they make it possible to study different aspects of visual functions. 

The visual evoked potential is the objective measurement of visual function monitored at the level of the occipital cortex with scalp electrodes. It is recorded with a uniform stimulus check size and a slow reversal rate throughout the field. 

This paper summarizes many of the studies pertaining to the significance of visual evoked potentials in the assessment of visual field defects in primary open-angle glaucoma. Included are the previous works related to the clinical utilization of VEPs for the objective assessment of typical visual field defects of POAG.

## 2. Challenges in the Past

The assessment of visual field defects with visual evoked potential has been a hard task. Ever since visually evoked cortical potentials were first used as a diagnostic aid the important question has been whether they could detect visual field defects. In earlier investigations [1], light-flash stimulators illuminating the entire retina were used and the bioelectrical responses from both hemispheres were compared. Because asymmetries between the hemispheres were also found in normal people only differences of 50 per cent or more between the responses of the right and left hemisphere were considered significant. 

Later, methods of stimulating the temporal and nasal parts of the retina separately with flash and checkerboard stimulation were introduced. Finally, a sophisticated method of separating the signals from retinal areas stimulated simultaneously was devised. However, there are few reports of the clinical application of these techniques. 

Since it is believed that elevation of intraocular tension causes pressure on the retinal nerve fibers bundles as they course into the optic nerve and results in the loss of visual function which is known to produce an alteration of the VEP waveforms; many earlier studies were interested in correlating VEP findings with perimetric defects. However, because the VEP is dominated by the central macular responses and reflects mainly macular function, it was altered only when the central visual field was disturbed [2].

Field defects with VEP have also been demonstrated using localized retinal stimulation [3–5]. The latter of the authors have described a technique for producing steady state visual evoked response (VER) to pattern reversal stimulation of retinal areas corresponding to discrete field quadrants. They arbitrarily classified their 21 patients with glaucomatous field defects into three categories according to the size of field defect namely—Early defects (occupying less than third of quadrant examined)—VERs from affected quadrant showed a large phase lag compared to normal homonymous quadrant. 

Moderate defects (occupying third to three quarters of affected quadrant)—either no VER was obtained on stimulation of retina corresponding to defective field quadrant or a phase delay was observed. 

Severe defects (three quarters to the whole quadrant tested)—In most cases, no VER was obtained from quadrants tested but in three cases small responses showing a phase shift were observed. They also conducted VER recordings with patient fixating centre of the 22° screen (full-field), the central 8° only and also the centre of the screen when the central 8° was occluded (peripheral). These tests did not show significant phase changes except in four cases with large field defects. The amplitude of the response from the eyes with moderate and severe defects was markedly reduced by comparison with that of the normal eye. Using this technique, an objective assessment of localized visual field defects was attempted, although it was based mainly on unilateral field defects.

Visually evoked cortical potentials were studied in six patients with a homonymous and six patients with a bitemporal hemianopia by presenting a pattern reversal stimulus separately to a temporal or nasal retinal area and by recording the responses from leads over the hemispheres [6]. Homonymous visual field defects were characterized by a reduction of VECPs from the affected hemisphere.

Both the topographical position and the dimension and degree of the diminished sensitivity of the visual field are important for changes in the evoked potentials, the nearer to the centre the visual field defect is localized, the larger the changes of the VEPs are expected to be. Thus a small relative scotoma located near the centre may affect significant changes on the VEP while a large absolute scotoma in the periphery may cause only minor changes in VEP [7].

The larger latency increments have been reported when measured in eyes with large field defects [8] but there was no direct relation between field size and latency. The visual field may be nearly intact with a definite increase of latency in the affected eye. 

Increased pattern VEP latency was significantly correlated with both the severity and location of visual field defects and the degree of cupping and pallor of the optic disc in another study [9].

However, some of the earlier works have demonstrated a poor sensitivity of the VEP to detection of CSG in patients with superior visual field defects due to the dominance of the inferior hemifield signal to the full-field VEP response [10].

Mean defect (MD) indicative of diffuse nonspecific nerve fibre loss correlated significantly (*P* < 0.05) in eyes with OAG in a previous study [11] where Flash evoked responses and the visual field indices (VFI) of the Octopus G1 program were recorded from 42 eyes of 21 patients, out of which 29 eyes had open-angle glaucoma (OAG) and 10 had ocular hypertension (OH).

The VEP changes observed by some authors in the form of prolonged P100 latency were consistent with the central visual field defects qualitatively and quantitatively [12]. Therefore, it was concluded that the latency of P100 can be a useful quantitative index in the evaluation of glaucomatous visual function damage. The difference in diagnostic sensitivity to glaucoma between VEP and visual field changes were studied and the authors have suggested that combination of the two may be a more useful index.

The pattern VEP was compared to the Octopus 2000R automated perimeter in the assessment of central visual function in chronic simple glaucoma (CSG) in 90 patients (52 males and 38 females) in two age bands 40–60 years and 61–80 years [13] VEP demonstrated a high detection rate (86.7%) with a relatively low false positive rate of 7.7% (*P* < 0.01). When the two tests were compared, absolute latency and field loss were poorly correlated but interocular differences showed much stronger correlation with the sum of field losses determined with static perimetry. This was true for both upper and lower hemifield testing. Thus once interindividual variability was eliminated; severity of field loss was mirrored by prolongation of VEP latency. 

### 2.1. Advent of Multichannel Pattern Visual Evoked Potentials

In recent years, multichannel pattern visual evoked potentials have begun to be compared to Humphrey perimetry in the assessment of central visual function in primary open-angle glaucoma. The multi-channel checkerboard reversal PVEPs waves to full-field and half-field stimulus of 25 normal persons and 74 patients with primary open-angle glaucoma were recorded and analyzed in a study [14]. All patients were examined using Humphrey field analyzer. The area of visual field corresponding to the area of retina stimulated during multi-channel PVEPs testing were analysed, straight-line correlation and regression analyses of the various multi-channel PVEPs parameters and the total dB losses were performed. The multi-channel PVEPs demonstrated that absolute latency and field loss were correlated in the late stage of glaucoma, and absolute amplitude and field loss were not correlated. The authors therefore inferred that in late loss of primary open-angle glaucoma, multi-channel PVEPs can provide a valuable, objective complement to Humphrey perimetry.

VEP measurements with presumable stimulation of single neuronal pathways can detect glaucomatous optic nerve damage in a considerable fraction of patients with visual field loss as glaucoma is associated with blue color vision disturbances [15]. They studied pattern VEP with colored stimuli to test blue sensitive pathway. Their study included 59 patients (96 eyes) with glaucomatous changes of the optic disc and visual field defects and 58 control eyes of 29 healthy subjects. Four types of pattern VEP stimulation (0.9 cycle/degree) were performed in all patients: achromatic, alternating sine-wave stripe pattern (activation of predominantly the magnocellular pathway), isoluminant, red-green stripe pattern (activation of predominantly the parvocellular pathway), and blue grating with yellow background adaptation (activation of the blue-sensitive pathway). In a paired correlation analysis with visual field defects, significant (*P* < 0.05) results were obtained with the perimetric MD value for all stimulations and with the neuroretinal rim area of the optic disc which again supports the validity of the VEP technique in glaucoma. Correlation coefficients were highest (*R* = 0.79, *P* < 0.01) for the peak time of the blue-yellow VEP. 

In spite of these results and the fact that there were no other confirmative reports about the usefulness of BY-VEP, there remains still uncertainty whether the Blue Yellow-VEP becomes pathologic before visual field or optic disc damages appear and whether it is able to predict these defects. 

To evaluate whether glaucomatous visual field defects could be related to an impaired retinal function, to a delayed neural conduction in postretinal visual pathways, or both; visual field by Humphrey perimeter (central 24-2 threshold test) and simultaneous recordings of visual evoked potential (VEP) and pattern electroretinogram (PERG) were assessed in 21 subjects with open-angle glaucoma (POAG) and in 15 age-matched controls [16]. VEP in POAG eyes showed significantly (*P* < 0.01) delayed P100 latency when compared with controls and correlated with mean deviation (index of global visual field damage, MD) (*P* < 0.001) and the P100 amplitudes were also significantly (*P* < 0.01) lower in POAG eyes than in control eyes and correlated with MD (*P* < 0.001). No significant correlations (*P* > 0.05) were found between electrophysiological parameters and corrected pattern standard deviation (CPSD), index of localized visual field damage, of 24-2 Humphrey Perimetry. Retinocortical time (RCT: difference between VEP P100 and PERG P50 latencies) and latency window (LW: difference between VEP N75 and PERG P50 latencies) were significantly (*P* < 0.01) longer in POAG eyes than in control eyes and correlated with MD (RCT: *P* < 0.001; LW: *P* < 0.001). He concluded that in patients with open-angle glaucoma the reduction of the index of global visual field damage (MD) could be ascribed to two sources of functional impairment: one retinal (impaired PERG) and one postretinal (delayed RCT and LW). In the postretinal impairment, a postsynaptic degeneration at the level of the lateral geniculate nucleus could be suggested.

To assess the presence of normal or abnormal pattern visual evoked potential (VEP) responses in patients with ocular hypertension and open-angle glaucoma (OAG), a study was performed on 80 normal control subjects, 68 ocular hypertension patients with intraocular pressure (IOP) < 18 mmHg under pharmacological treatment and 84 OAG patients with intraocular pressure (IOP) < 18 mmHg under pharmacological treatment [17] VEPs using high-contrast (80%) 15′ checkerboard stimuli with 2 reversals per second were recorded. They showed highly significant positive correlation (*P* < 0.001) between P100 amplitude and HFA24/2 MD and MD values in their POAG patients were negatively correlated with the latency time of P100.

### 2.2. Advent of Multifocal Visual Evoked Potentials

With the multifocal technique, visual evoked potentials (VEPs) can be recorded simultaneously from many regions of the visual field in a matter of minutes. Recently, the multifocal visual evoked potential technique (mfVEP) has generated considerable interest, especially among those seeking objective measures of glaucomatous damage. If both eyes of an individual are normal, then mfVEPs recorded for monocular stimulation of each eye are essentially identical. However, the amplitude and waveform of the mfVEP responses vary across individuals, as well as across the visual field within an individual. These variations are related to cortical anatomy and to the cortical sources contributing to the mfVEP. The mfVEP is predominantly generated in V1. Although there are undoubtedly extrastriate contributions, these contributions are probably smaller for the mfVEP than for the conventional VEP. The mfVEP is not a small version of the conventional VEP. 

To determine the relationship between spatially localized multifocal visual evoked potentials (mfVEPs) and Humphrey visual fields (HVFs) in patients with unilateral field defects, Humphrey visual fields and mfVEPs were obtained from 20 patients with unilateral field losses due to either ischemic optic neuropathy or glaucoma [18]. Monocular mfVEPs were obtained for each eye. The amplitude of the mfVEP responses was calculated and estimates of the HVF loss in the same regions of the field used for the mfVEP were obtained by interpolating the 24-2 HVF data. Their results showed that monocular mfVEP amplitude decreased with HVF loss, although small mfVEP signals were not uniquely associated with poor fields. On average, the monocular mfVEP was indistinguishable from noise for field losses between −5 and −10 dB, and good monocular mfVEP amplitudes were never associated with extensive visual field loss. The interocular ratio of the mfVEP amplitudes correlated well with the difference between the HVF values of the 2 eyes.

The monocular and interocular results were consistent with a linear relationship between the amplitude of the signal portion of the mfVEP response and linear HVF loss. One way to produce this relationship would be if both the signal in the mfVEP and linear HVF loss were linearly related to the percentage of local ganglion cells lost.

To detect ganglion cell damage with the mfVEP requires methods for analyzing the responses and for displaying the results. A method for detecting ganglion cell damage has been described [19]. This method compared the monocular responses from the two eyes of an individual and produced a map of the defects. This map is in the form of a probability plot similar to the one used to display visual field defects measured with automated perimetry. Procedures were described for directly comparing these mfVEP probability plots to the probability plots for Humphrey visual fields (HVFs). 

Using the techniques described therein, the relationship between the amplitude of the mfVEP and the sensitivity loss of the HVF was discussed. The evidence supports a simple model in which the amplitude of the signal portion, but not the noise portion, of the mfVEP response is proportional to HVF loss where HVF loss is expressed in linear, not dB, units. 

It was hypothesized that both the signal in the mfVEP, and the sensitivity of the HVF, are linearly related to ganglion cell loss. A theoretical approach was developed which allowed a direct comparison of the efficacy of the mfVEP and HVF in detecting glaucomatous damage. 

In short, when the mfVEP has a large SNR it will often be superior to the HVF in detecting damage. On the other hand, when the mfVEP has a small SNR, the HVF will probably be superior. 

In summary, the authors concluded that the mfVEP has a place in the clinical management of glaucoma, although it is not likely to replace static automated achromatic perimetry in the near future. However, this is an evolving technology and the future will undoubtedly see major improvements in the mfVEP technique. The multifocal VEP (mfVEP) technique is still in infancy and there are as yet no studies to determine its reliability compared with other methods of investigation. 

Another study was conducted to compare latencies of conventional visual evoked potentials (cVEPs) and multifocal VEPs (mfVEPs) in the same patients [20] 75 eyes (47 patients), 75 eyes with suspected glaucoma (46 patients), and 41 control eyes (22 subjects) underwent achromatic automated perimetry and mfVEP and cVEP testing. The mfVEP stimulus was a scaled dart board with 60 sectors; each sector was a pattern-reversing checkerboard. The cVEP stimulus was a reversing checkerboard with checks of either 15 minutes or 60 minutes in width. They have shown that the latency of both the mfVEP and cVEP (conventional VEP) bore no obvious relationship to the mean deviation of the visual field in their study. 

### 2.3. Advent of Colour Pattern Visual Evoked Potentials

To investigate the changes of color pattern reversal visual evoked potential (CPR-VEP) of primary glaucoma using different temporal frequencies CPR-VEP was recorded using Vision Monitor visual electrophysiograph at different temporal frequencies (1, 2, 4, 8, 16, and 32 Hz) and different color stimulations (black/white, red/green, blue/yellow) in 41 cases (70 eyes) with primary glaucoma (glaucoma group) and 13 normal subjects (26 eyes) (normal control group) [21] P100 wave amplitudes were compared. In the normal control group, P100 amplitudes declined while the temporal frequency of black/white stimulation was increasing, but they had peaks at 2 Hz and 8 Hz red/green stimulation and blue/yellow stimulation. In the glaucoma group, CPR-VEP P100 declined while temporal frequency was increasing fewer than 3 color stimulations, but had a peak at 8 Hz. At 2 Hz–16 Hz, P100 amplitudes were related with the mean defect of Humphrey visual field, especially with all 3 color stimulations at 8 Hz and with blue/yellow stimulation at 2 Hz and 16 Hz. P100 amplitude was most different under the 3 color stimulations between the 2 groups at 8 Hz. The authors concluded that the changes of CPR-VEP P100 amplitude can objectively reflect the glaucoma visual function damage. CPR-VEP P100 amplitude has certain value in studying glaucoma under different color stimulations (black/white, red/green, blue/yellow) at 8 Hz, and blue/yellow stimulation at 2 Hz and 16 Hz.

To investigate the difference in color pattern reversal visual evoked potential (CPR VEP) between primary open-angle glaucoma (POAG) and primary angle closure glaucoma (PACG) patients CPR-VEP were obtained in 17 eyes of 12 POAG patients, 56 eyes of 41 PACG patients, and 26 eyes of 13 age-equivalent normal persons at an ascending series of temporal frequency (1, 2, 4, 8, 16, and 32 Hz), and color stimulation (black/white, red/green, and blue/yellow) [22] P100 wave amplitudes and latencies of these patients were compared, respectively, with those of the normal group. With black/white stimulation, the P100 wave amplitudes were reduced with the increase of temporal frequency in the 3 groups. The P100 wave latencies were extended with the increase of temporal frequency with different color stimulations. The P100 amplitudes were PACG group > normal group > POAG group and black/white > blue/yellow > red/green. The P100 wave latencies in the POAG group and the PACG group were extended compared with the NC group, but there was no significant difference between PACG group and POAG group. Thus they concluded that P100 amplitude of PACG is higher, and POAG is lower than normal. The P100 wave latencies of PACG and POAG are extended.

A very recent study was undertaken to evaluate the color Doppler imaging (CDI) and pattern visual evoked potential (P-VEP) examinations in primary open-angle glaucoma (POAG) patients and investigate the relation between flow velocities measured by CDI and P-VEP examination in POAG patients [23], 65 POAG patients, and 45 control subjects were investigated for CDI evaluation of the ophthalmic artery (OA), short posterior ciliary artery (SPCA) and central retinal arteries (CRA), and the latency and amplitude of P100 in P-VEP were recorded. The differences of CDI and P-VEP parameters among POAG and control groups were compared. The latency of P100 in VEP delayed and the amplitude of P100 decreased in the POAG patients comparing with that of the control group. They have found the MD values in the POAG patients were negatively correlated with the latency time of P100, which was agreed with the previous studies. 

With an attempt to assess the correlation of visual field indices with VEP parameters in primary open-angle glaucoma, we conducted a study on a larger cohort of 100 POAG patients and 200 control subjects of central Indian population [24]; we observed that our POAG patients showed different degrees of visual field impairment detected by a reduction in mean defect (MD) and by an increase in pattern standard deviation (PSD). The reduced MD observed in our POAG patients was significantly correlated with the abnormal cortical electrophysiological responses. There was a good significant negative correlation of P100 latency and MD and a significant relationship of N155 latency and P100 duration. This finding of significant correlation between the values of MD and those of VEP parameters is consistent with the results reported in other studies in which abnormal VEP responses were related to visual field defects assessed by Goldmann perimetry [2, 11, 13, 25, 26], or by static perimetry [11, 13]. Our results are also in close agreement with a recent study [23] which has documented that the MD values in POAG patients were negatively correlated with the latency time of P100, which also corroborates the findings of previous workers [17]. The mean VEP P100 amplitude of POAG patients in our study was highly significantly (*P* < 0.001) correlated with value of mean defect (MD) in dB. Our results correspond with recent study [23] which reported that the MD values in their POAG patients were positively correlated with the amplitude of P100. Our results also concur with the findings of previous workers [17] who have put forth similar conclusion.

## 3. Conclusion

From this paper, it can be concluded that VEP is an important visual electrophysiological tool which has been used for the evaluation of visual field defects in primary open-angle glaucoma. It is endowed with an added advantage of objectivity. The electrophysiological test like VEP is a more objective measure of optic nerve function because it is not influenced by cognitive factors or the motor skills of the subject as compared with the psychophysical tests. Further significant correlations between the magnitude of the VEP latencies and the size of visual field defect and optic disc cupping or pallor over the years confirm the validity of VEP method in primary open-angle glaucoma. Further, the correlation obtained by us between all the electrophysiological VEP parameters and MD of Humphrey static perimetry suggests that the impaired visual cortical responses observed in glaucoma patients can be revealed by both electrophysiological and psychophysical methods. In addition, the severity of global glaucomatous damage evidenced by reduction in MD could depend on the delay in neural conduction from retina to the visual cortex as revealed by the significant correlation between VEP latencies and MD.
